# Analysis of polarized-light effects in glass-promoting solutions with applications to cryopreservation and organ banking

**DOI:** 10.1371/journal.pone.0199155

**Published:** 2018-06-18

**Authors:** Prem K. Solanki, Yoed Rabin

**Affiliations:** Biothermal Technology Laboratory, Department of Mechanical Engineering, Carnegie Mellon University, Pittsburgh, PA, United States of America; Michigan State University, UNITED STATES

## Abstract

This study presents experimental results and an analysis approach for polarized light effects associated with thermomechanical stress during cooling of glass promoting solutions, with applications to cryopreservation and tissue banking in a process known as vitrification. Polarized light means have been previously integrated into the cryomacroscope—a visualization device to detect physical effects associated with cryopreservation success, such as crystallization, fracture formation, and contamination. The experimental study concerns vitrification in a cuvette, which is a rectangular container. Polarized light modeling in the cuvette is based on subdividing the tridimensional (3D) domain into a series of planar (2D) problems, for which a mathematical solution is available in the literature. The current analysis is based on tracking the accumulated changes in light polarization and magnitude, as it passes through the sequence of planar problems. Results of this study show qualitative agreement in light intensity history and distribution between experimental data and simulated results. The simulated results help explaining differences between 2D and 3D effects in photoelasticity, most notably, the counterintuitive observation that high stress areas may correlate with low light intensity regions based on the particular experimental conditions. Finally, it is suggested that polarized-light analysis must always be accompanied by thermomechanical stress modeling in order to explain 3D effects.

## Introduction

Cryopreservation by vitrification (*vitreous* in Latin means *glassy*) is considered to be the only promising technique for long-term preservation of large-sized tissues and organs [1,2], with direct implications on the future of biobanking and transplant medicine [3]. Vitrification is facilitated by loading the tissue with cryoprotective agent (CPA) solutions. The CPA is characterized by an exponential increase in viscosity with the decreasing temperature when cooled fast enough, such that the CPA reaches extremely high viscosity in a shorter period of time than the typical time scale to form and grow crystals. Below some temperature threshold, known as the glass transition temperature (*T*_*g*_), the viscosity is so high, and hence molecular mobility is so slow, that the material can be considered solid for practical applications. When the specimen is loaded with CPA and exposed to such conditions, the biological material is trapped in a glassy state [4] and can potentially be stored for an indefinite period of time.

Suppressing ice crystallization [2,5] is only one of the requirements for successful cryopreservation by vitrification, while other key phenomena that affect successful specimen recovery from cryogenic preservation are associated with the natural degradation of the biological material with temperature, CPA toxicity [6], specimen preparation and handling, and thermomechanical stresses that are driven by the cryopreservation protocol [7–11]. These thermomechanical stresses may devastate the structural integrity of the specimen, in a process which is the focus of the current study. (In this study, thermomechanical stress and thermal stress are used interchangeably.)

Thermal stress is driven by the phenomenon of thermal expansion, either due to one or more of the following effects: (i) phase transition in the specimen, where pure water expands upon freezing [12]; (ii) temperature gradient across the specimen, where different layers of the material may tend to contract at different rates [13]; (iii) thermal expansion mismatch between the specimen and the container, imposing mechanical stress on the specimen [14]; and (iv) any combination of the above effects [7–11]. As a result, thermal stress may develop during specimen cooling to cryogenic storage, during thermal equilibration at cryogenic storage or at even higher temperatures to relax stresses, and during rewarming the specimen from cryogenic storage [7,13].

The phenomena associated with thermal stress can be more easily controlled in small samples, in a typical size ranging from μm and mm, with cryopreservation success examples relating to stem cells [15,16], embryos [17], pancreatic islets [18], and corneas [19,20]. Thermal stress can be reduced by increasing the CPA concentration and, thereby, reducing the cooling rate needed to suppress ice crystallization and growth. However, this tradeoff may come at the expense of increased toxicity potential to the specimen, which affects its viability and functional recovery. Such a strategy may prove sufficient when the mechanical function of the specimen has a higher priority than the biological function, with heart valves as example [21]. Successful cryopreservation by vitrification of larger-size samples, measured in cm or larger, remains an outstanding challenge. Making cryopreservation of larger specimens a practical reality calls for an integrated approach to solve the multiple-parameter optimization problem [22].

Cryomicroscopy has been used extensively for visualization of physical events during cryopreservation in micro-samples [23–27]. Unfortunately, the translation of microscale observations to criteria for cryopreservation successes for macroscale specimens is frequently impractical [9]. With these observations in mind, cryomacroscopy has been invented for *in-situ* visualization of macroscale physical events [9]. In this context, four types of cryomacroscopes have been presented in the past decade [8–10,28,29]. The most recent and advanced type is the scanning cryomacroscope, which incorporates a mechanism to capture movies on physical events that are larger than the field of view in any single still image [29]. Data gathered from scanning cryomacroscopy experiments is used for the analysis of photoelasticity in the current study.

The scanning cryomacroscope was designed to retrofit the lid of a commercially available controlled-rate cooler, which is commonly used in cryopreservation facilities. This setup permits the observation of physical effects during practical cryopreservation protocols. In order to further enhance the observed effects, the principles of a plane polariscope were incorporated with the scanning cryomacroscope [30]. It has been demonstrated that polarized light can be a powerful means for detection of contamination, crystal formation, fracture formation and even strains in the specimen [30,31]. The analysis of the visualized mechanical strain by means of polarized-light cryomacroscope is the focus of the current study, which is related to the phenomenon of photoelasticity [32].

Photoelasticity was first reported by David Brewster in 1815 [33,34], and is based on the principle of double refraction (birefringence); discovered by Erasmus Bartholinus in 1669 while experimenting with Iceland crystals [35]. Over the years, photoelasticity has been extensively used for experimental stress analysis in 2D, using relatively thin specimens [36–39]. Development of automated polariscopes has been instrumental in development of 3D photoelasticity [40]. Various methods such as scattered-light photoelasticity [41], frozen-stress technique [42], integrated photoelasticity (tensor-field tomography) [43], and optical slicing [44], have been reported for the investigation of stresses using 3D photoelasticity. Today, portable polariscopes are commercially available for stress analysis of transparent objects in 3D [45].

The current study integrates the investigation of photoelasticity with cryomacroscopy, which represents a complicated system to analyze due to system limitations. Here, the photoelasticity effects are created in a confined space, in the extreme environment of a cryogenic controlled-rate cooler. In this processes, the stress in the specimen is not only dependent upon the instantaneous temperature, but also upon the thermal history, which results in a unique path of mechanical loading. The resulting 3D stress field is non-uniform, which creates an integrated effect on the polarized light travelling through the medium. The aim in the current study is to develop a computational framework for the evaluation of polarized-light experimental observations, created in the unique environment of cryopreservation by vitrification. This framework facilitates the analysis and data interpretation of previously reported polarized-light effects [30].

## Materials and methods

### Experimental apparatus

**Fig 1:**
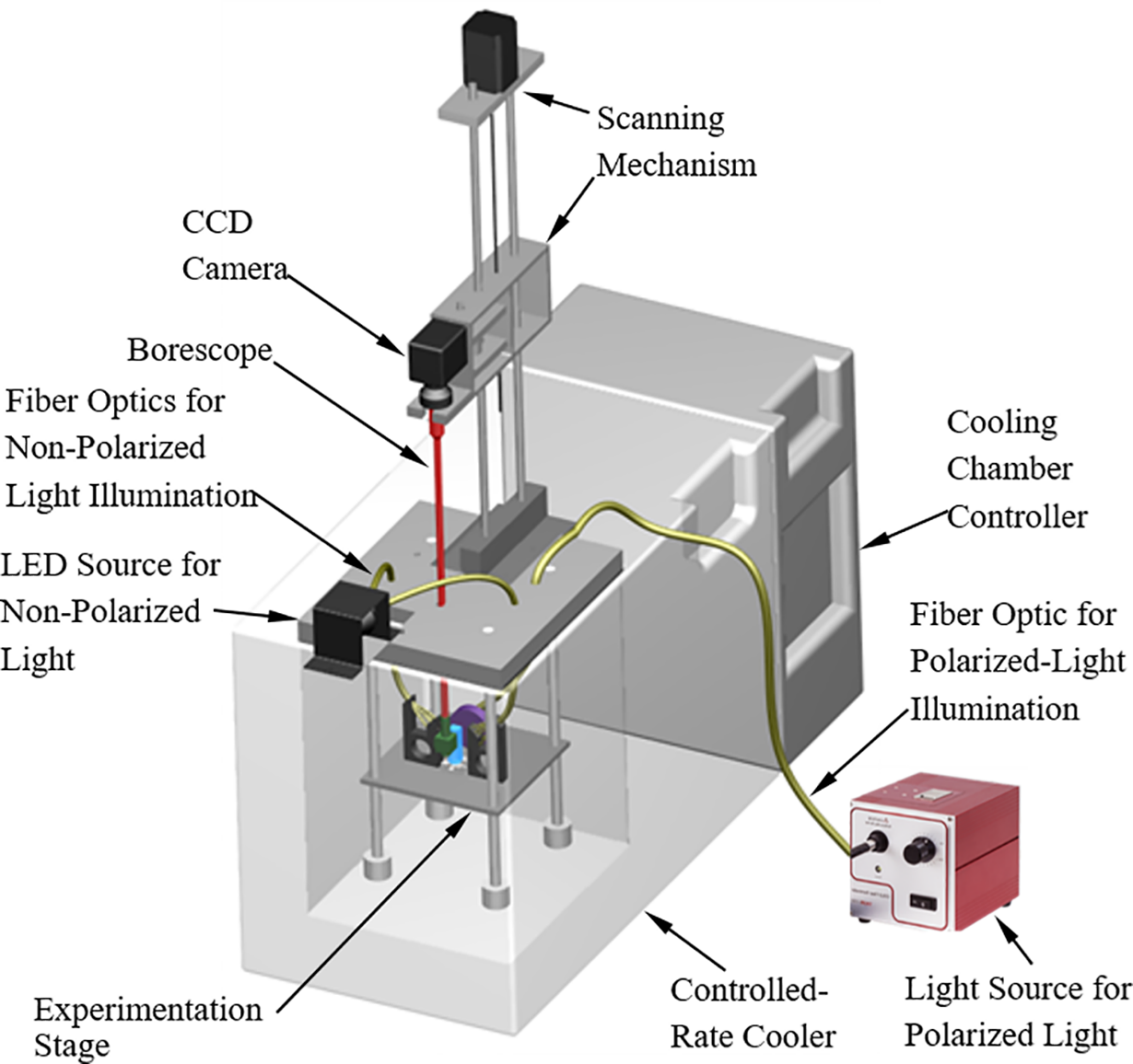
Schematic illustration of the scanning cryomacroscope setup incorporating polarized-light means. Via an integrated graphical user interface, independent computerized means (not shown) are used to control the scanning mechanism of the cryomacroscope, to streamline images from the CCD camera, and to log temperature data from strategically located sensors. The cooling chamber is independently controlled by a commercial programmable controller. A more detailed setup is presented in [30].

**Fig 2:**
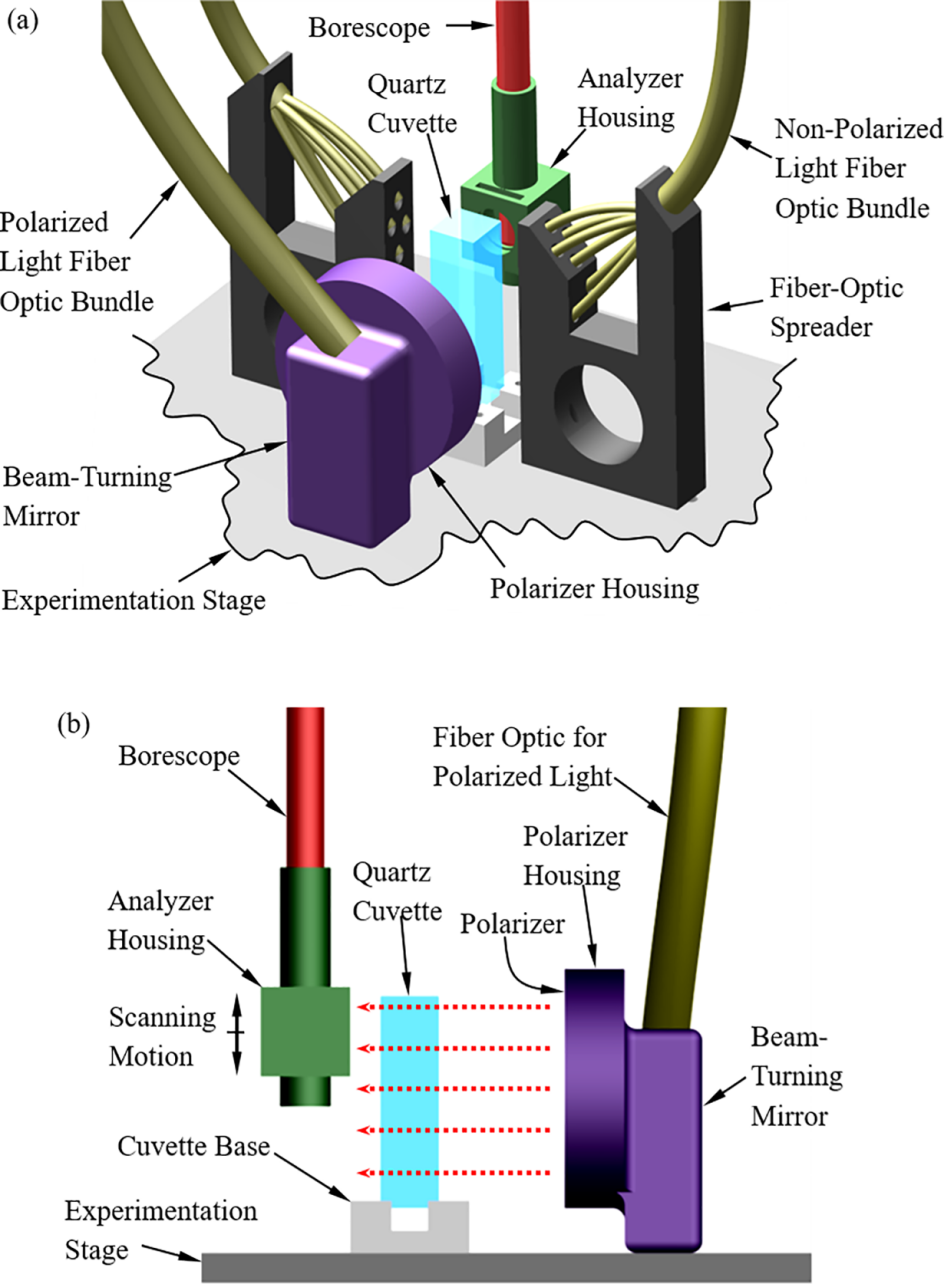
Schematic illustration of the experimentation stage of the scanning cryomacroscope, incorporating polarized-light means. (a) Isometric view including all illumination components. (b) Side view highlighting the light polarization and filtration components. where the red-dashed arrows represent the direction of polarized light illumination. (Reprinted from Cryobiology, 73(2), Feig, J. S. G., Eisenberg, D. P., and Rabin, Y., Polarized light scanning cryomacroscopy, part I: Experimental apparatus and observations of vitrification, crystallization, and photoelasticity effects, pp. 261–271, Copyright (2016), with permission from Elsevier).

The scanning cryomacroscope, Fig 1 [29] and its add-on polarized-light setup, Fig 2 [30], have been presented previously and are presented here in brief only, for the completeness of presentation. Due to the harsh environment surrounding the sample and space limitations, all electronic components and mechanisms are placed externally to the cooling chamber. Light is delivered by various fiber-optics bundles, while the image of the specimen is captured via a borescope (Hawkeye HH2992, Gradient Lens Corporation, Inc., NY, USA).

The cryomacroscope comprises of the following key elements: (i) a commercial cooling chamber and controller (Kryo 10–16 controlled by Kryo 10–20, Planer PLC, UK); (ii) an experimentation stage (Fig 2); (iii) a high-speed, light-sensitive, CCD camera, connected to the external end of the borescope; (iv) a scanning mechanism, comprising a stepper motor, a controller, and a carriage system; (v) an array of T-type thermocouples for monitoring the thermal history via a computerized data acquisition system (not illustrated); (vi) an LED light source and fiber-optics bundles to provide diffuse light illumination onto the sample; (vii) a polarized-light source, a dedicated fiber-optics bundle, a mirror, and filters, to provide polarized-light conditions (Fig 2A); and (viii) a proprietary cryomacroscope control code (C^3^) to control the various cryomacroscope components, real-time monitoring of images and temperatures, data streamlining, and post-processing of a digital movie for each experiment, overlying time and temperature data.

While different transparent containers and vials can contain the specimen (Fig 2), the current study uses a cuvette (essentially a rectangular vial) due to its superior optical properties and minimal specimen distortion. The current study focuses on a quartz cuvette for cryogenic temperatures (FireflySci Inc., NY, USA), having dimensions of 12.5 mm × 12.5 mm × 45 mm. The cryogenic stage is designed to alternate between non-polarized light and polarized light on demand. With reference to Fig 2A, the non-polarized light is directed perpendicular to the field of view, which was found to best enhance effects such as fractures and crystallization. The fiber-optics spreader has been designed and 3D-printed (ABS) for illumination at 45° to the cuvette surface, in order to further prevent reflections from the cuvette outer surfaces.

To facilitate the polarized-light investigation, light was delivered from an external halogen-light source (150W at 3200K, Thorlabs Inc., NJ, USA), through a high-quality fiber-optics bundle (91 cm in length, 6.4 mm in diameter; Core Fiber Bundle, Thorlabs Inc., NJ, USA) to an aluminum housing of a silver-coated, beam-turning mirror (97.5% reflectivity, Thorlabs Inc., NJ, USA). The mirror directs the light perpendicular to the polarizer (50 mm in diameter), illuminating the cuvette in the direction of the red-dashed arrows in Fig 2B. An analyzer (12.5 mm in diameter) has been retrofitted to the tip of the borescope by means of a 3D-printed (ABS) housing. Note that the borescope setup includes an internal 45° mirror to reflect the polarized light into the axial direction of the borescope. Both polarizer and analyzer are made of a 0.3 mm-thick dichroic polarizing film sheet (>99% efficiency, Thorlabs Inc., NJ, USA), sandwiched between two protective glass windows. Each window has an antireflective coating which is optimized for the visible part of the spectrum (0.4 μm to 0.7 μm).

### Geometric model

**Fig 3:**
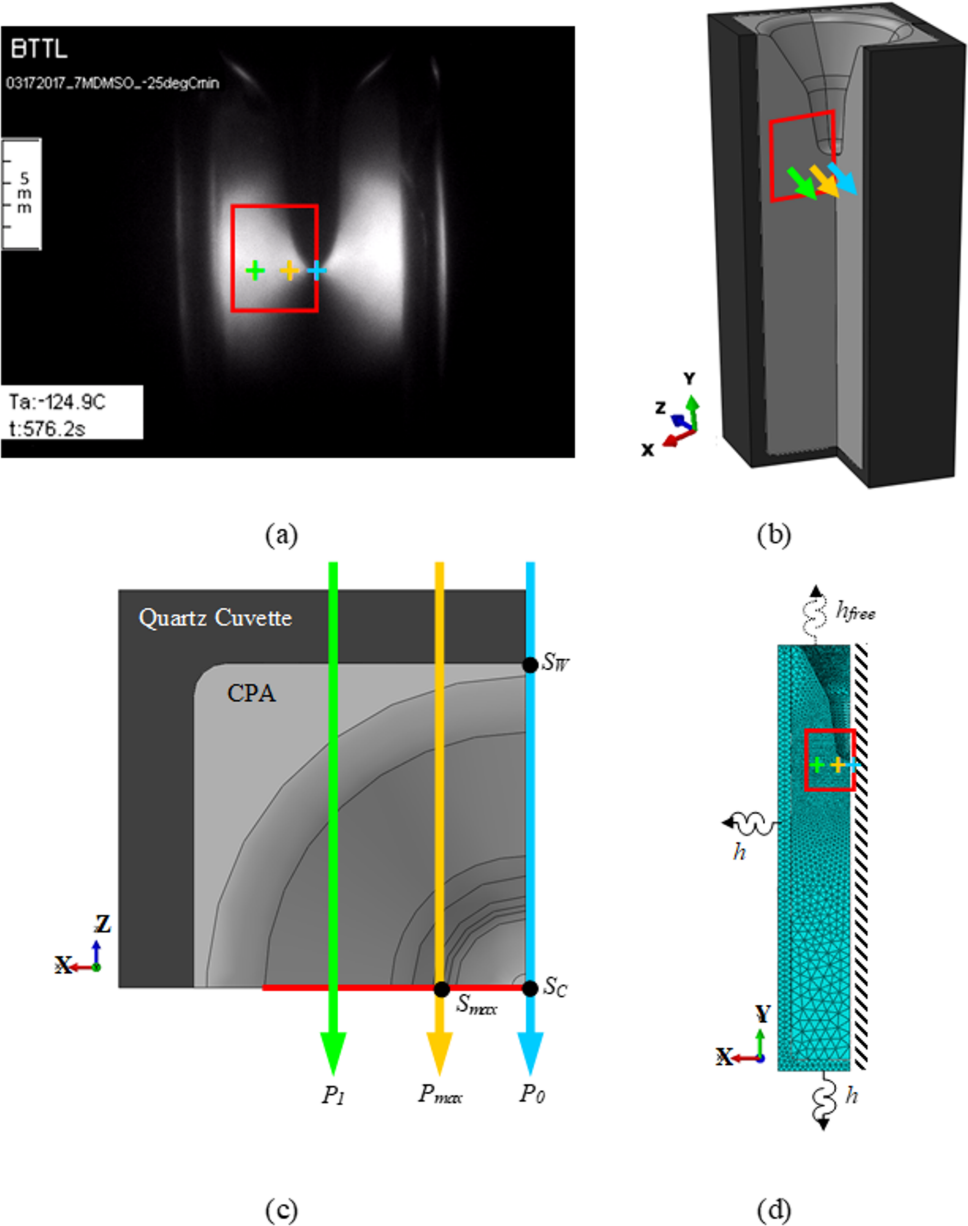
Geometric model and frame of reference for polarized light investigation. Presentation of three representative paths of light, *P*_0_, *P*_1_, and *P*_*max*_ (colored arrows) and a frame of reference for light refraction analysis (a red rectangle, measures 4 mm in width and 5 mm in height): (a) a snapshot from a polarized-light cryomacroscopy movie (side view of the cuvette); (b) schematic illustration of the reconstructed geometric model; (c) top view of the cross section used for *f*_*𝜎*_ parametric estimation, where S_C_ and S_W_ represent virtual temperature sensors located on *P*_0_, at the center of the domain and at the CPA-wall interface, respectively, while *S*_*max*_ represents virtual stress sensor located on *P*_*max*_ at the symmetry line; and (d) an FEA mesh for thermal-stress analysis using ABAQUS, highlighting the boundary conditions used for heat transfer formulation, where *h* is the overall heat transfer coefficient accounting for forced convection and radiation heat transfer on the outer walls of the cuvette, while *h*_*free*_ accounts solely for heat transfer by free convection. Note that the frame of reference starts 1 mm away from the wall, to avoid optical reflection effects around the fillet at the inner walls corner of the cuvette.

Fig 3A displays a representative polarized-light image of the CPA surface after it is cooled to an intermediate hold temperature above the glass transition temperature. The cavity at the center of the domain is caused by the effect of thermal contraction with the decreasing temperature [7,29–31], and can be explained as follows. As the cooling process progresses inwards, the CPA contracts more than the cuvette due to its higher thermal expansion coefficient. At higher temperatures, the CPA is free to flow and the level of CPA gets lower to compensate for the difference. At some point along the thermal history, the first CPA layer adjacent to the wall becomes so cold and, thus, its viscosity becomes so high that it cannot easily flow anymore. From this point onwards, that layer gains solid-like characteristics. Any additional CPA tendency to contract can now be compensated by either the remaining low-viscosity CPA at the center, by mechanical stress in the solid-like region, or by a combination of both. This process repeats itself layer by layer, with an increasing level of differential contraction, which is compensated by a decreasing amount of free-to-flow CPA. In this process, a bottleneck-like type cavity is created and, eventually, the entire CPA domain becomes solid-like.

Experimental observations by means of polarized light reveal that the cavity formation described above is driven by very low mechanical stresses, which are negligible when compared with the high mechanical stress magnitude that leads to fracture and structural damage—the focus of the current study. These observations can be explained by the fact that the viscosity of the vitrifying material changes by 12 orders of magnitude, as the material is cooled from room temperature to cryogenic-storage temperature. Only when the viscosity of the material is high enough to inhibit flow in a practical time scale, significant stresses can build up. These experimental observations are consistent with previous experimental and theoretical studies [8,46], as well as with the design of commonly accepted cryopreservation protocols.

Following the above observations, the vitrification process is simplified to have two primary stages according to the stress level: free-of-stress large deformations (in practice, deformation under negligible stress), and significant stress which is associated with small deformations. This approach enables decoupling the heat transfer problem from the solid mechanics problem in the second stage, where results of the heat transfer solution serve as input for the solid mechanics problem. Consistently, the geometry of the cavity for the analysis in the second stage can be directly extracted from cryomacroscopy images.

A sample reconstruction of the shape of the bottleneck-like cavity from cryomacroscopy movies is illustrated in Fig 3B. This cavity leads to stress concentration effects and its shape is associated with the formation of fractures [31]. Hence, the analysis in the current study focuses on the tip of this cavity and its vicinity, where the red rectangle in Fig 3 represents the frame of reference for polarized-light calculations. Excluded from the analyzed region is a thin layer of material behind the fillet, at the inner walls corner shown in Fig 3C, which causes optical distortion. Due to the 4^th^ order rotational symmetry along the cuvette centerline, thermal stress is simulated in only one quarter of the domain. While the actual geometry of the cavity varies with the thermal protocol, the current analysis focuses on one representative thermal protocol for the computation framework presentation, while comparing experimental data with simulation results. For the purpose of the current discussion, three representative horizontal light paths are illustrated in Fig 3, located on an *x-z* plane at a distance of 0.5 mm below the tip of the cavity: *P*_0_ passes through the center of the cuvette’s cross-section, *P*_1_ passes at a distance of 3 mm from its center, and path *P*_*max*_ passes at a distance of 1.3 mm from its center, where the subscript *max* signifies maximum simulated light intensity for the light path.

The thermomechanical problem was solved using the finite element analysis (FEA) commercial code ABAQUS (Dassault Systems, Inc), using 10-noded quadratic tetrahedron elements (DC3D10 for heat transfer analysis and C3D10H for stress analysis) with the FEA mesh displayed in Fig 3D. Prior to meshing, internal light paths were defined and discretized, which facilitated nodes sharing of the neighboring elements along the light paths, to simplify light refraction calculations as discussed below. Based on a mesh convergence study, the maximum element size in the direction of light propagation was limited to 0.2 mm, while a coarser mesh was selected outside the frame of interest, with a maximum element length of 1.5 mm in the CPA domain. For the cuvette, the maximum element size in the direction of light propagation was limited to 0.4 mm, while the maximum element length in other directions was limited to 0.7 mm. Automatic time stepping was selected for the solution of the thermomechanical problem in ABAQUS, with the following limits at any given time step: maximum temperature change of 1°C, maximum inelastic strain rate of 1%, and maximum time step size is limited to 25 seconds.

### Heat transfer formulation

The heat transfer problem is assumed to be independent of the solid mechanics problem, where the solution of the former serves as input for the latter [7,31,47]. The CPA domain is modeled as a modified Maxwell fluid with a temperature dependent viscosity [7,47]. In the temperature range of interest for stress development (below -110°C for 7.05M DMSO, which is the tested CPA in the current study), the viscosity is high enough for the CPA to be considered as a solid for all practical purposes. Hence, heat transfer within the CPA domain is assumed to be governed by conduction only:

$$C\dot{T}=\nabla ∙(k\nabla T)$$ (1)

where *C* is the volumetric specific heat, *T* is the temperature, *k* is the thermal conductivity, and the dot represents a time derivative.

The temperature-dependent volumetric specific heat in the CPA, *C*, is assumed to follow the Einstein model for internal energy storage [31]:

$$C=\frac{3Nk_{b}}{M_{u}}{\left(\frac{\theta_{E}}{T_{a}}\right)}^{2}exp\left(\frac{\theta_{E}}{T_{a}}\right){\left[exp(\frac{\theta_{E}}{T_{a}}-1)\right]}^{-2};\;\theta_{E}=\frac{\hbar \omega }{\kappa_{b}}$$ (2)

where *N* is the number of oscillators, *k*_*b*_ is the Boltzmann constant (1.38×10^−23^ J/K), *M*_*u*_ is the molecular weight, *T*_*a*_ is the absolute temperature, *θ*_*E*_ is the Einstein temperature, ℏ is the reduced Planck’s constant (1.054×10^−34^ J.s), and *ω* is the frequency of oscillation of the molecule (6.415×10^13^ Hz) [31]. An effective temperature-dependent thermal conductivity is assumed within the CPA domain [31], which takes into account free convection effect at temperatures above -60°C (Table 1).

**Table 1:** Material properties of the CPA (7.05M DMSO) and quartz cuvette used in this study.

Property	Material				
	DMSO 7.05 M			Quartz Cuvette	
Viscosity, Pa·s	1.77×10^4^ 2.8190×10^−27^ e^-0.6447 *T*^ 4.06×10^14^	-106°C≤T -143°C≤T<-106°C T≤-143°C	[48]	N/A	
Glass transition temperature, °C	-132		[49]	N/A	
Density, kg/m^3^	1100		[50]	2655–0.75 *T*	[51]
Thermal conductivity, W/m °C	0.899 + 1.01×10^−2^ *T* 0.312 + 2.54×10^−4^ *T*	-60°C≤*T*≤25°C -170°C≤*T*≤-60°C	[31]	6.57 + 0.016 *T*	[52]
Specific heat, J/kg °C	Eq (2)		[31]	692.89 + 1.852 *T*– 0.0031 *T*^*2*^ + 4.902×10^−6^ *T*^*3*^	[53]
Thermal Expansion coefficient, 1/°C	1.1×10^−4^		[50,54–56]	7×10^−7^	[57]
Young’s modulus, GPa	0.8		[58,59]	72	[60]
Poisson’s ratio	0.25		[7]	0.17	[60]

Heat transfer in the cuvette walls is assumed by conduction, using Eq (1), with the temperature-dependent properties listed in Table 1. No contact resistance to heat transfer is assumed between the CPA and the cuvette.

A combined effect of convection and radiation heat transfer is considered between the cuvette boundary and the cooling chamber environment [31]:

$$-k\frac{dT}{d\hat{n}}=h\left(T_{c}-T_{\infty }\right)$$ (3)

where $\hat{n}$ is the direction normal to the cuvette wall, *h* is overall heat transfer coefficient (measured as 346 W/m^2^°C in the current setup [31]), and the subscripts *c* and ∞ refer to the cuvette wall and the air temperature within the cooling chamber, respectively. The free convection at the CPA-cooling chamber interface (upper surface of the CPA, Fig 3D), has been shown negligible in a previous study [31]. The thermal protocol for the air temperature in the cooling chamber is discussed in detail in the thermal protocol section below.

### Solid mechanics formulation

In the CPA domain, the total strain rate in the Maxwell fluid model is calculated as [8,48,58]:

$${\dot{\varepsilon }}_{total}={\dot{\varepsilon }}_{creep}+{\dot{\varepsilon }}_{elastic}+{\dot{\varepsilon }}_{thermal}$$ (4)

The creep, elastic, and thermal strain rates are calculated by:

$${\dot{\varepsilon }}_{creep}=\frac{S}{2\eta }$$ (5)

$${\dot{\varepsilon }}_{elastic}=\frac{1}{E}\left[\left(1+\nu \right)\dot{\sigma }-\nu I\cdot tr\left(\dot{\sigma }\right)\right]$$ (6)

$${\dot{\varepsilon }}_{thermal}=\beta \dot{T}I$$ (7)

where ***S*** is the deviatoric stress tensor, η is the viscosity, *E* is the elastic modulus, υ is the Poisson ratio, ***I*** is the identity matrix, *tr* is the trace matrix, and *β* is the linear thermal expansion coefficient.

Linear elastic behavior is assumed in the cuvette walls with the specific mechanical properties listed in Table 1. The CPA is modeled to adhere to the inner surface of the cuvette walls during vitrification. This behavior is formulated via a *tie constraint* in ABAQUS simulations. Consistent with the discussion in the Geometric Modeling section, zero stress distribution is assumed as an initial condition for the mechanical problem.

### Polarized light formulation

Light refraction in the medium is modeled using the stress-optic relationship [61]:

$$\delta =t_{S}\lambda \frac{\sigma_{1}-\sigma_{2}}{f_{\sigma }}$$ (8)

where *δ* is the relative retardation of light of wavelength *λ* as it passes through a material of thickness *t*_*S*_ under planar stress conditions; *σ*_*1*_ and *σ*_*2*_ are the planar principal stresses and *f*_*σ*_ is the fringe constant.

Since Eq (8) represents light refraction in a planar problem (i.e., a 2D case), the 3D domain is subdivided into a series of thin layers, stacked perpendicular to the direction of light propagation, where light refraction in each layer is approximated as in a planar problem. The secondary principal stresses [61] for each layer are calculated from the simulated thermomechanical process, *t*_*s*_ is a geometrical parameter of the problem (determined from the convergence studies discussed below), and the fringe constant is found experimentally using a parametric estimation method (also discussed below). Light refraction through each layer is calculated separately and sequentially along the specific path of light propagation, where the output light from one layer is the input into the next. The quality of the above approximation is discussed below.

The cryomacroscopy color images have been converted to gray-scale images for the purpose of the numerical analysis. Here, the intensity of the three light components as captured by the CCD camera (red, green, and blue) has been averaged to obtain a single value at each point on the measured image, with Fig 3A as an example for the compiled gray-scale image. Since the current experimental observations are based on not more than one fringe, and since each light component within this fringe range show the same trend of intensity increase with the increasing strain [62], the above averaging approach bears no effect on the following discussion and conclusions. If more than one fringe would be observed, then a monochromatic filter would have to be added to the cryomacroscopy setup in order to create a similar effect in preparation for image analysis.

#### Parametric estimation of the fringe constant in the CPA

Each path of light passes through two materials: the cuvette and the CPA. While the fringe constant of the quartz cuvette is available in the literature (*f*_*σ*_ = 180 MPa·mm [63]), a parametric estimation technique was employed to find the fringe constant in the CPA domain as follows. Using ABAQUS for meshing, each analyzed path of light is divided into 25 linear elements in the CPA domain and additional 3 linear elements in the cuvette wall, each coinciding with the edges of one thermo-mechanical element. Each of these 25 CPA light elements is further subdivided into *n* parallel slices for a light refraction convergence study. The stress tensor in each slice is interpolated from the thermal-stress solution at the nodal points.

The number of slices, *n*, and the fringe constant *f*_*σ*_ are optimized sequentially for the best match between the simulated light intensity at the end of the light path of interest and the measured intensity during experimentation at the same locations. First, the simulated light intensity was found to exponentially approach a constant value with the increasing *n* value, where an *n* value of 5000 was found to affect the overall intensity by less than 1%. Next, a least-square parametric estimation technique for *f*_*σ*_ was employed within the CPA domain, by minimizing the following target function:

$$F=\sum_{i=1}^{l}{\left(I_{e,i}-I_{s,i}\right)}^{2}$$ (9)

where *l* is the number of light paths in the *x-z* plane, and *I*_*e*_ and *I*_*s*_ are the experimental and simulated intensities of light at the same location, respectively.

#### Light refraction in a planar case

**Fig 4:**
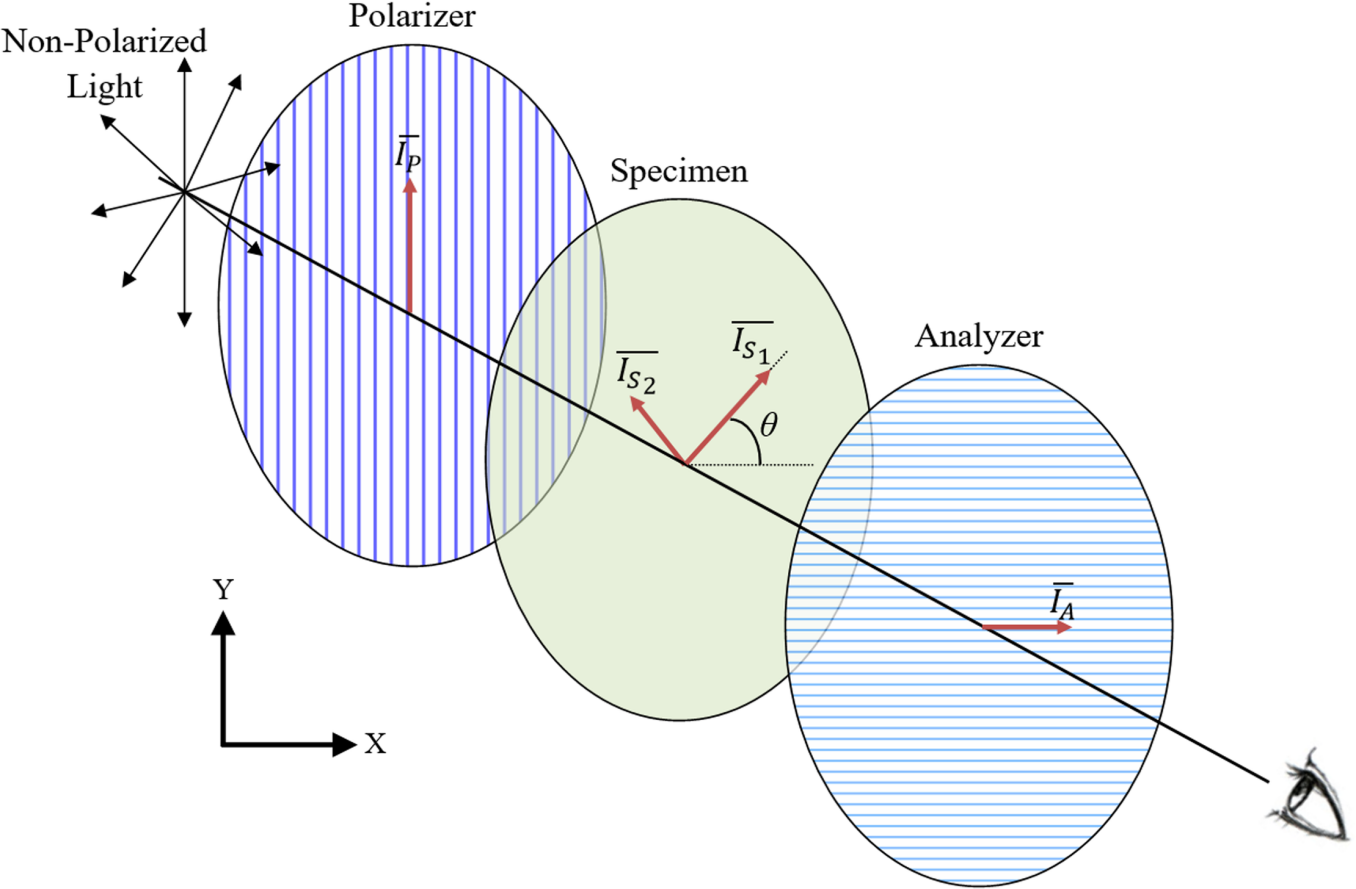
Schematic illustration of a plane polariscope. Where $\bar{I_{P}}$ is the light entering the birefringent specimen, $\bar{I_{S_{1}}}$ and $\bar{I_{S_{2}}}$ are the fast and slow components for light, respectively, as they pass through the specimen, and $\bar{I_{A}}$ is the observed light passing through the system.

Analysis of light refraction in the planar case (Fig 4) is presented first in brief (for more detail see [61]). Next presented is a framework for the analysis of a 3D object constructed of multiple layers, where each layer is modeled as a planar problem. With reference to Fig 4, the incoming light vector passing through the polarizer is given by [64]:

$$\bar{I_{P}}=ae^{i\frac{2\pi }{\lambda }\left(z-ct\right)}$$ (10)

where *a* is the light amplitude, *c* is the speed of light, and *λ* is the wavelength.

In a planar stress problem, the frame of reference (coordinate system) on the *x*-*y* plane can be rotated about the *z* axis by an angle *θ* such that the shear stress vanishes [65]. The stresses with respect to this rotated frame of reference are defined as the principal stress *σ*_*1*_ and *σ*_*2*_ (׀*σ*_*1*_׀ > ׀*σ*_*2*_׀):

$$\theta =\frac{1}{2}tan^{-1}\left(\frac{2\sigma_{xy}}{\sigma_{x}-\sigma_{y}}\right)$$ (11)

where *σ*_*x*_, *σ*_*y*_ and *σ*_*xy*_ are the components of the stress tensor before rotation. Light refraction of the incoming light vector $\bar{I_{P}}$ is dependent upon the principal stresses and, hence, this vector is subdivided into two corresponding and orthogonal components:

$$\bar{I_{P_{1}}}=\bar{I_{P}}sin\theta \;;\;\bar{I_{P_{2}}}=\bar{I_{P}}cos\theta$$ (12)

The birefringence theory states that the refractive indexes of the above components will differ in the direction of the principal stresses [35]. Consequently, these light components will travel through the specimen at different speeds, resulting in a faster moving component $\bar{I_{S_{1}}}$ and a lagging behind component $\bar{I_{S_{2}}}$ (Fig 4). The phase lag between these components is given by:

$$\Delta_{l}=\frac{2\pi \delta }{\lambda }$$ (13)

Consistently, the two components of the light vector in the direction of principal stresses leaving the same layer are given by:

$$\bar{I_{S_{1}}}=\bar{I_{P_{1}}}\;;\;\bar{I_{S_{2}}}=\bar{I_{P_{2}}}e^{i\Delta_{l}}$$ (14)

where the subscript *S* refers to the layer surface farthest from the light source.

Finally, the light vector to reach the analyzer is given by:

$$\bar{I_{A}}=\bar{I_{S_{1}}}cos\theta -\bar{I_{S_{2}}}sin\theta$$ (15)

where the negative sign signifies that the components of $\bar{I_{S_{1}}}$ and $\bar{I_{S_{2}}}$ parallel to the direction of polarization of the analyzer are in opposite directions, as illustrated in Fig 4. Substituting $\bar{I_{S_{1}}}$ and $\bar{I_{S_{2}}}$ from Eq (12) into Eq (15) yields:

$$\bar{I_{A}}=\bar{I_{P}}sin\theta cos\theta -\bar{I_{P}}e^{i\Delta_{l}}cos\theta sin\theta =\bar{I_{P}}sin\theta cos\theta (1-e^{i\Delta_{l}})$$ (16)

However, an observer standing behind the analyzer will only see the light intensity, which is proportional to the square of the light amplitude [61]. The light intensity field is normalized in the current analysis, where the observed intensity becomes:

$${\left|\bar{I_{A}}\right|}^{2}=a^{2}{sin}^{2}2\theta {sin}^{2}\frac{\Delta_{l}}{2}$$ (17)

#### Light refraction in 3D

Recall that each beam of light is subdivided into *m* = 25 linear elements in the CPA domain, which are further divided into *n* = 5000 layers, where calculations of light refraction in each layer follows the above planar formulation. In this multilayer expansion, the calculated refracted light leaving one layer is taken as the input light entering the next layer, farther away along the path of light. Accordingly, $\bar{I_{S_{1_{k}}}}$ and $\bar{I_{S_{2_{k}}}}$ are the faster and slower components of the light vector leaving the *k*^th^ layer, respectively:

$${\left[\begin{array}{c} \bar{I_{S_{1}}}\\ \bar{I_{S_{2}}} \end{array}\right]}_{k}=\left\{ \begin{array}{cc} {\left[\begin{array}{cc} 1 & 0\\ 0 & e^{{∆}_{l}} \end{array}\right]}_{k}R_{\theta_{k}}\left[\begin{array}{c} 0\\ \bar{I_{P}} \end{array}\right] & k=1\\ {\left[\begin{array}{cc} 1 & 0\\ 0 & e^{{∆}_{l}} \end{array}\right]}_{k}R_{\theta_{k}}R_{\theta_{k-1}}^{-1}{\left[\begin{array}{c} \bar{I_{S_{1}}}\\ \bar{I_{S_{2}}} \end{array}\right]}_{k-1} & k=2..n\times m \end{array}\right.$$ (18)

where *R* is a 2×2 rotation matrix. Therefore, the light vector components leaving a 3D model subdivided to *n×m* layers is given by:

$${\left[\begin{array}{c} \bar{I_{S_{1}}}\\ \bar{I_{S_{2}}} \end{array}\right]}_{n\times m}=\left(\prod_{k=1}^{n\times m}{\left[\begin{array}{cc} 1 & 0\\ 0 & e^{{∆}_{l}} \end{array}\right]}_{k}R_{\theta_{k}}R_{\theta_{k-1}}^{-1}\right)\left[\begin{array}{c} 0\\ \bar{I_{P}} \end{array}\right]$$ (19)

Similar to Eq (15) for the planar model, the light vector to reach the analyzer after passing the 3D case is given by:

$$\bar{I_{A}}=\bar{I_{S_{1_{k}}}}cos\theta_{k}-\bar{I_{S_{2_{k}}}}sin\theta_{k}\;;\;k=n\times m$$ (20)

### Tested materials

The current study focuses on dimethyl sulfoxide (DMSO), which is a key ingredient in many CPA solutions for cryopreservation by vitrification [54]. In particular, this study focuses on 7.05M DMSO, which has proven a good reference solution for thermal stress studies [54]. Table 1 lists the relevant material properties used in the current analysis.

### Thermal protocol

**Fig 5:**
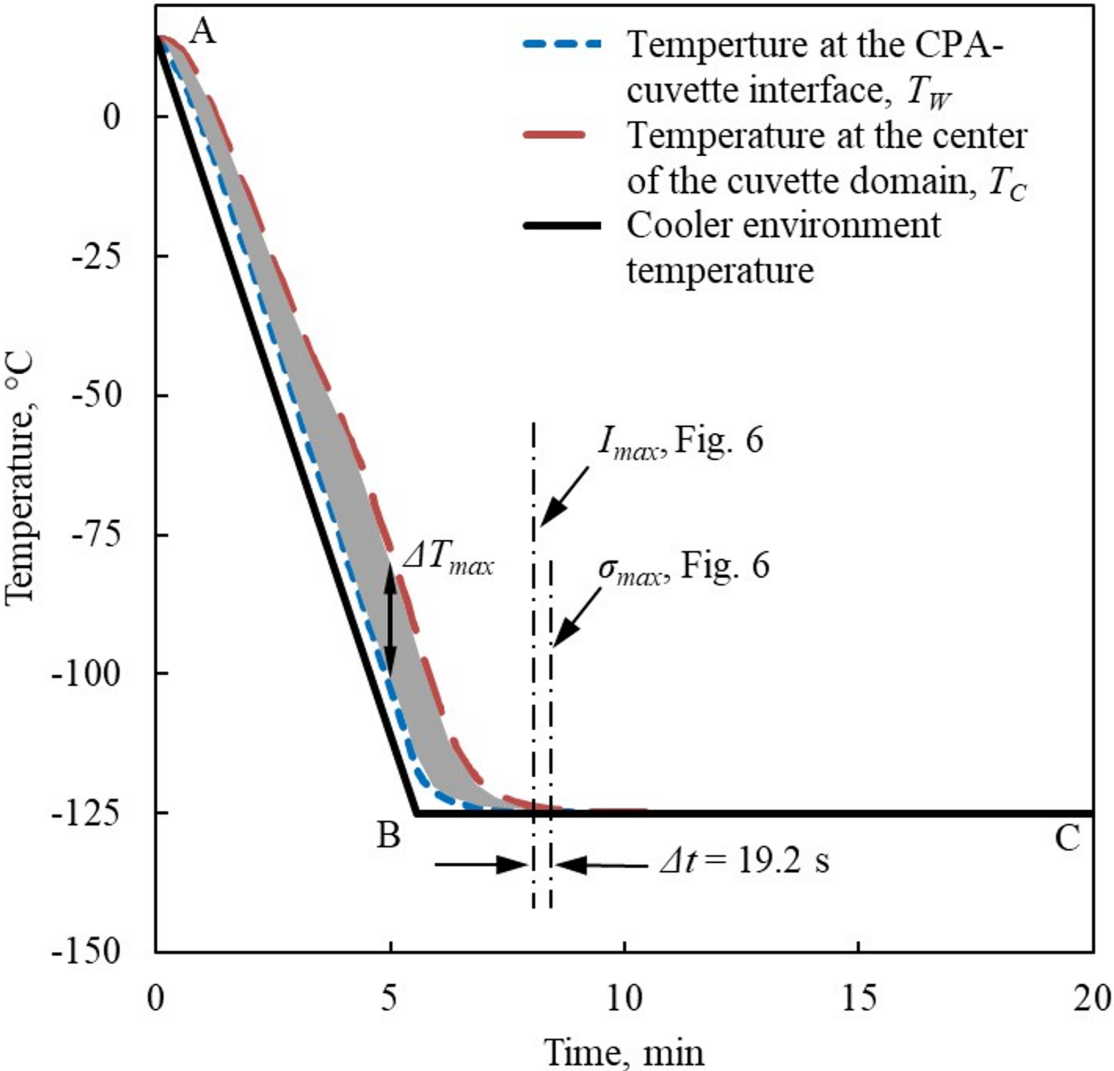
Thermal history of the environment and strategically selected points in the cuvette shown in Fig 3B. The instances at which maximum first principal stress, *σ*_*max*_, and simulated normalized maximum light intensity, *I*_*max*_, are highlighted according in Fig 6.

This study focuses on the cooling part of the cryogenic protocol in order to validate the proposed polarized-light model and calculation framework. Fig 5 displays the experimental thermal histories *T*_*W*_ and *T*_*C*_ at two locations along path *P*_0_ (Fig 3A) at the CPA-cuvette interface, *S*_*W*_, and at center of the cuvette, *S*_*C*_, respectively. The temperature difference between these thermal histories is reflective of the overall temperature distribution within the CPA domain. Initially, the cuvette containing the CPA is held inside the cooling chamber, until it reaches thermal equilibrium at 14°C. With reference to Fig 5, this cooling protocol is based on constant cooling rate from the above initial temperature down to -125°C (segment A-B), followed by a constant temperature hold at the minimum temperature (segment B-C). The glass transition temperature of 7.05M DMSO is about -132°C [8], while the temperature hold at -125°C is designed to facilitate stress relaxation [7]. At that hold temperature the material is viscous enough to support mechanical stress for a short period of time, but its viscosity is low enough to permit the stress to creep away over a longer period of time, measured in minutes. While a stress relaxation stage in such a short temperature interval from the glass transition temperature range is not commonly practiced, it has been demonstrated theoretically [7,13] and experimentally [29–31] to reduce the thermal stress at cryogenic storage. This temperature hold permits thermal equilibration after the fast cooling rates to avoid crystallization at higher cryogenic temperatures and before cooling is restarted at a much lower rate, to prevent structural damage at cryogenic storage.

**Fig 6:**
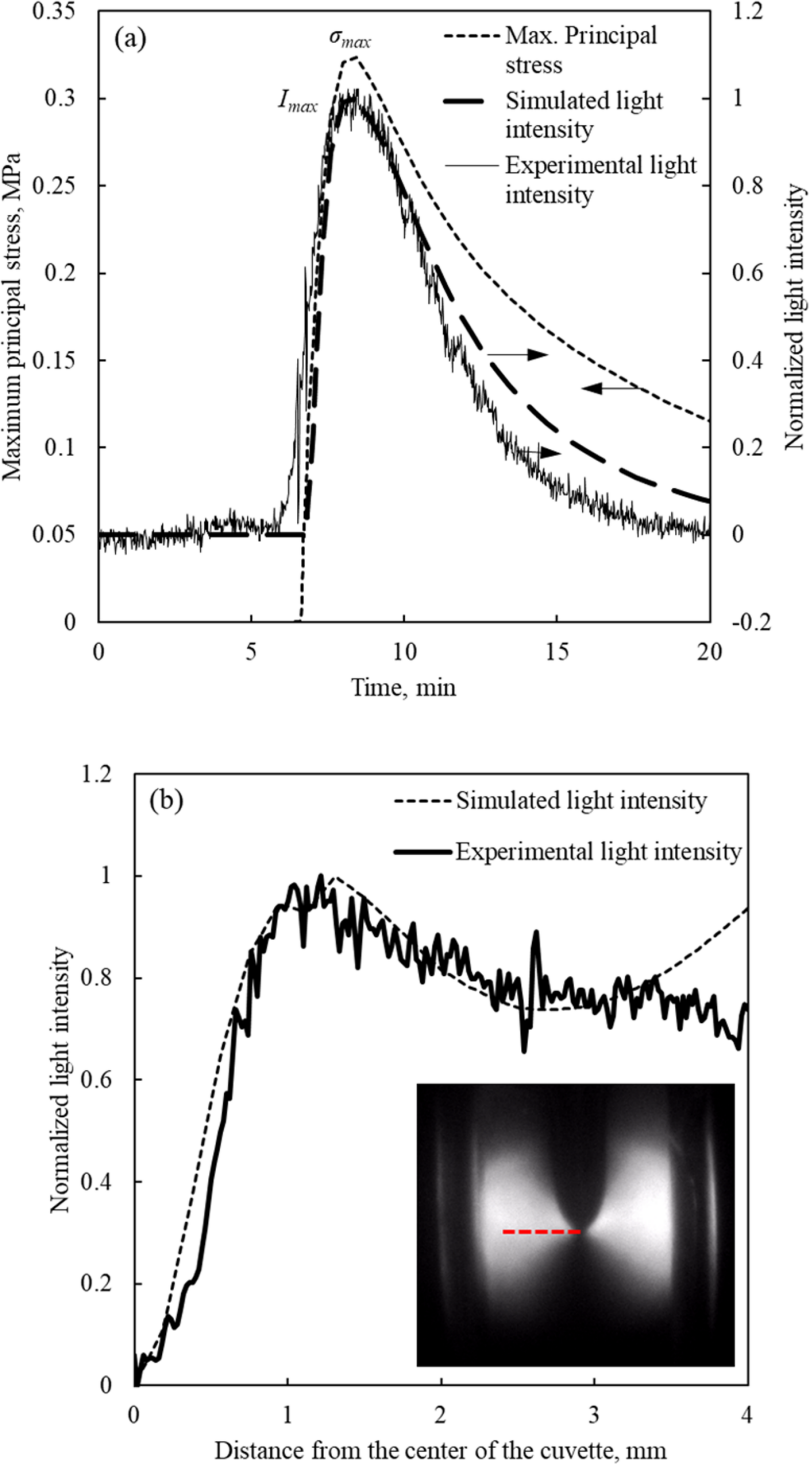
Comparison of experimental data and simulated results for normalized light intensity and maximum principle stress. (a) Experimentally measured light intensity history at *S*_*max*_ location (see Fig 3C), in comparison with the calculated maximum principal stress, *σ*_1_, history and the simulated light intensity at the same location. (b) Variation of light intensity with the distance from the center of the cuvette at 0.5 mm below the tip of the cavity (along the dotted line in the inset), at the instance when maximum light intensity is observed (*t* = 505 s).

### Polarized light protocol

In principal, the polarizer—filtering the outgoing light from the illumination source, and the analyzer—filtering the light after passing the specimen, must be oriented perpendicular to one another, with undetermined dependency upon their absolute orientation. Three polarizer-analyzer couple setups were tested in the current study: (A) analyzer parallel to the major axis of the cuvette, (B) analyzer perpendicular to the major axis of the cuvette, and (C) analyzer oriented at 45°, midway between cases A and B. Following the underlying principles of a plane polariscope, the polarizer was oriented perpendicular to the analyzer in all cases.

The polarizer light source was set to 60W for all experiments, which enabled continuous light measurements while avoiding CCD sensor saturation, where software auto adjustment of the capture image was disabled for all the experiments [30].

## Results and discussion

For light refraction calculations, 131 light paths are selected within the frame of reference illustrated in Fig 3 as follows. The horizontal and vertical distance between adjacent light paths in the *x-y* plane are ^1^/_6_ mm and 1 mm, respectively. The higher resolution in the horizontal direction facilitates parametric estimation for *f*_*σ*_ at a higher certainty (Eq (9)). Furthermore, the vertical distance between adjacent light paths along the centerline of the cuvette is decreased to 0.25 mm for finer intensity analysis. The cavity at the center of the cuvette was excluded from this analysis, due to the inability of separating light reflection from light refraction effects at its surface. Polynomial approximation was used to compute the light intensity at the edges of the region of interest, where element edges are not aligned with the light paths. A built-in MATLAB (Mathworks, Inc.) subroutine was used for scattered data interpolation in order to create a continues intensity field within the frame of reference; this subroutine uses a triangulation-based interpolation method [66].

Parametric estimation performed on a horizontal cross-section, 0.5 mm below the cavity tip (where the paths of light are illustrated in Fig 3), yielded *f*_*σ*_ = 6.7×10^−3^ ± 1.4×10^−4^ MPa·mm for the CPA. Data from all the light paths coinciding with this cross-section where used for the analysis. This particular cross section was selected since the maximum light intensity was observed on it, with peak value at *t* = 505 s. Fig 6 displays the comparison of the experimental and simulated intensity of light using the optimized value for *f*_*σ*_.

**Fig 7:**
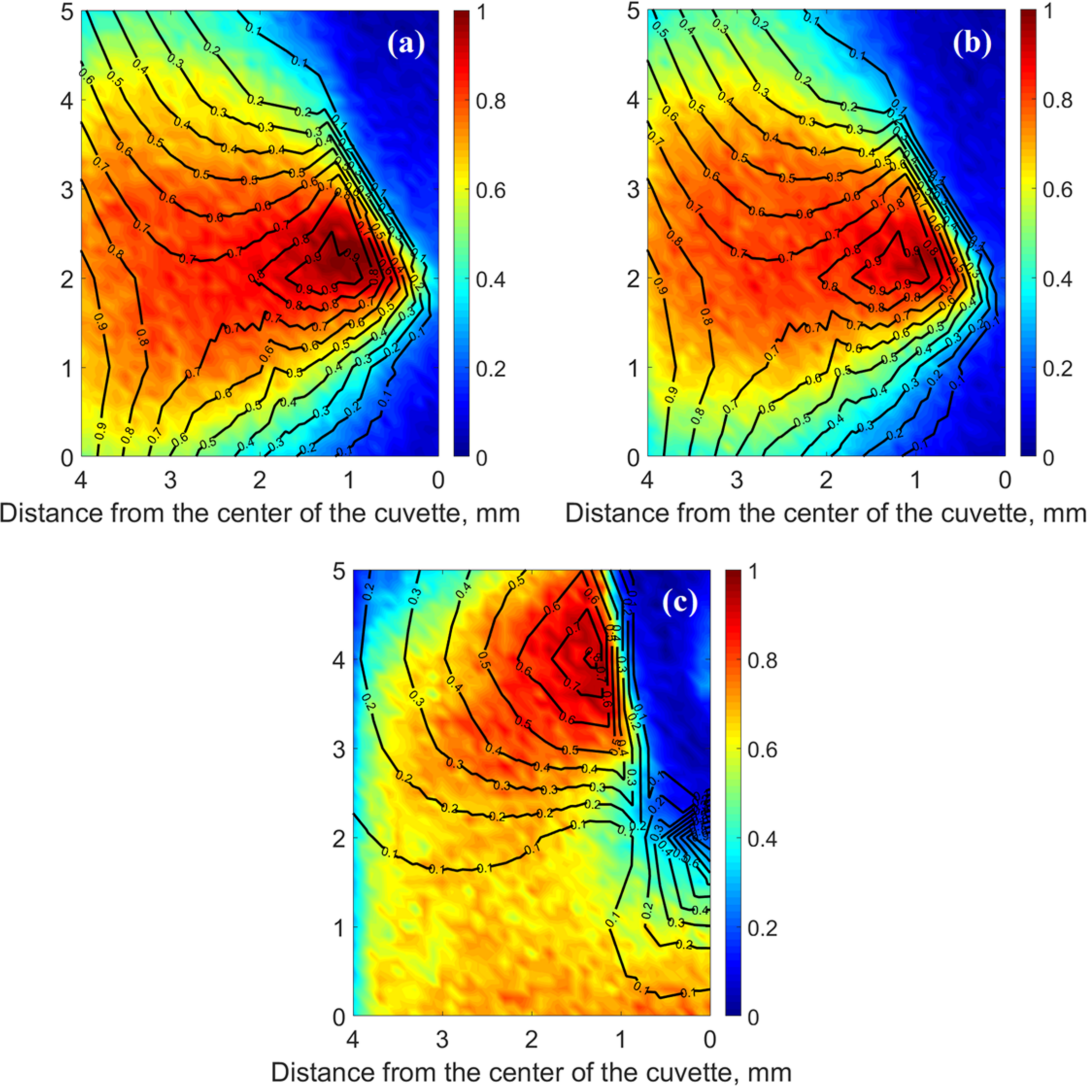
Color-coded comparison of experimental data and simulation results for the normalized gray-scale light intensity field. The gray-scale compiled light intensity field from experimental data is color-coded, where red represents high intensity and blue represents low intensity. The simulated light intensity field is represented by black contour lines, where 1 represents maximum intensity. (a) The analyzer is oriented parallel to the centerline of the cuvette. (b) The analyzer is oriented perpendicular to the centerline of the cuvette. (c) The analyzer is oriented at 45° − mid-angle between Cases A and B. This comparison is related to the field strain that develops after cooling the chamber at a rate of 25°C/min from room temperature, and subsequent temperature hold at -125°C for 170 s.

Fig 6A displays a light intensity comparison between measured data and simulated results as they vary along the experiment. The light intensity history is calculated along *P*_*max*_, where the simulated light intensity is found to be maximum (Fig 7A). In the absence of a specific time stamp to reference the light intensity recording with the thermal protocol, the experimental light intensity is shifted by 66 s in the specific analysis, to align the maxima of the experimental data and simulated light intensities. While a detailed system description and analysis have been presented previously [29,30], the need for the above time shift is described herein in brief for the completeness of presentation. Three key units of the cryomacroscope are off-the-shelf electronic systems, which do not share the same internal clock: the temperature data logger, the thermal controller of the cooling chamber, and the video recorder. Since these units are manually triggered along the specific cryogenic protocol, time alignment between the corresponding datasets is essential. Fig 6A also displays the first principal stress history (σ_1_, which is the largest principal stress out of the three) at location *S*_*ma*x_ (Fig 3C) for comparison. Fig 6B displays the light intensity as a function of distance from the center of the cuvette at light paths 0.5 mm below the tip of the cavity (on the same *x*-*z* plane as of *P*_0_, *P*_1_, and *P*_*max*_, Fig 3).

The stress history resulting from a similar thermal history has been discussed previously [7,13], with the most relevant observation that the developed stress is insignificant under constant cooling rate conditions even when the CPA gradually becomes highly viscous. At higher temperatures, when the viscosity is very low, the CPA is free to flow, which does not permit stress buildup. At lower temperatures along the A-B segment in Fig 5, when the entire domain cools at a uniform rate, insignificant differential thermal expansion across the domain is the reason for why thermal stress does not buildup, despite the fact that the viscosity gradually becomes significant. Only when the temperature starts to equilibrate below the glass transition temperature and after the material has already gained solid-like characteristics, significant thermal stress develops. The result is residual stress at cryogenic storage, a phenomenon which is directly related to the fact that different layers in the CPA have gained solid-like behavior at different times [14].

When a temperature hold period above but close to glass transition is added, such as the -125°C in the current study (recall that *T*_*g*_ = -132°C), the CPA is viscous enough to allow stress to rise, but the viscosity is not high enough to sustain stress for an extended period of time. As a result, the stress will creep away (or relax) over time from the maximum value *σ*_*max*_ shown in Fig 6A [7,13]. A consistent trend is observed for the normalized light intensity curve in Fig 6, which increases to significant values after the CPA has reached the same temperature-hold stage, and subsequently decays as the material undergo stress relaxation.

**Fig 8:**
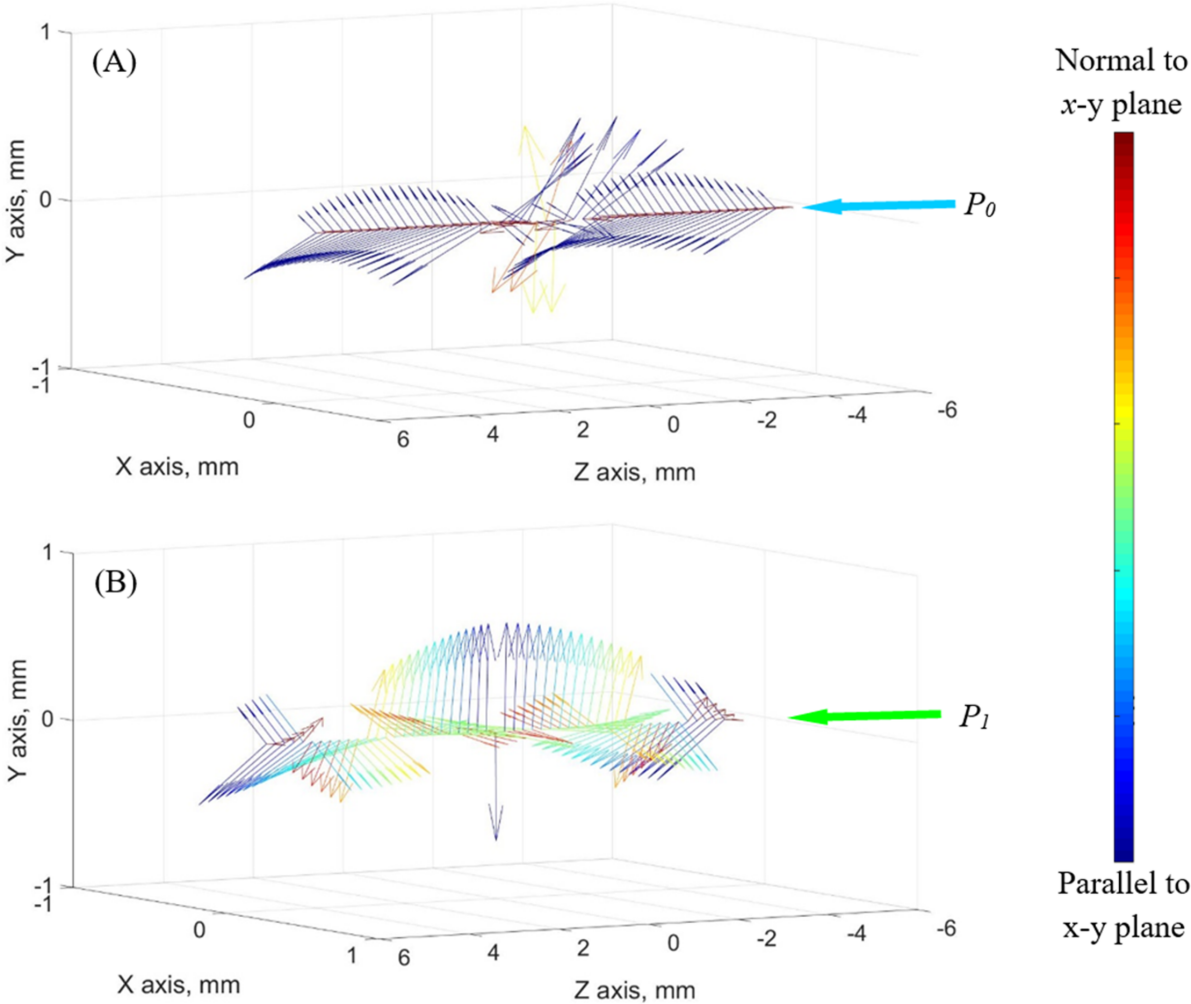
Principal stresses along representative paths of light. Principal stresses along the representative paths of light from Fig 3 (a) *P*_0_ and (b) *P*_1_, where the arrows are proportional to the stress magnitude, while the color scheme corresponds to the relative orientation of the stress vector with respect to the path of light: blue represents a stress vector perpendicular to the path of light while red represents a stress vector parallel to it.

In order to correlate the above events with the thermal history, Fig 5 displays the time at which the first principal stress reaches a maximum value, *σ*_*max*_, at location *S*_*max*_, and the time at which the simulated light intensity along the path of interest (the path passing through point *P*_0_) reaches a maximum value, *I*_*max*_. The time lag between *σ*_*max*_ and *I*_*max*_ is 19.2 s for the specific experimental conditions. In considering this time lag, one should bear in mind that while the light intensity is an accumulated effect along the path of view, the maximum principle stress is a localized affect at a discrete point. Since these effects are inherently different, there is no reason to believe that they must occur at the same instant, although they are expected to happen in a close time window. Finally, the agreement between simulated and measured normalized light intensities deteriorates towards the wall of the cuvette (3.3 mm <), which is attributed to changes in the direction of the principal stresses, as discussed in detail in the context of Fig 8 below.

Fig 7 displays the comparison of the normalized light intensity between experimental data and simulated results, at the instant at which the simulated intensity reaches a maximum value (t = 505 s). This comparison is qualitative only, since the light intensity is normalized, while the maximum intensity in the experimental setup is dependent upon various parameters, such as the light source, its power, mirror quality, and the quality of the light reflector; calibration for these parameters is deemed impractical.

Results from three polarizer-analyzer couple setup are displayed in Fig 7. Fig 7A compares the experimental and simulated light intensity fields in Case A, where the polarizer is parallel to the centerline of the cuvette. The following observations can be made in Case A: (i) the experimental light intensity above the tip of the cavity is essentially nonexistent due to the presence of the cavity; (ii) a region of low intensity is observed below the tip of the cavity, despite the fact that stresses are maximal within this region—this is a counterintuitive outcome when the results are compared with the planar case; and (iii) the light intensity decreases with the horizontal distance from the tip of the cavity, although simulation results suggest faster decay than experimentally observed. The qualitative agreement is stronger near the center of the cuvette, and it diminishes towards the wall of the cuvette.

Fig 7B displays a similar comparison between experimental measurements and simulated light intensity in Case B, where the orientation of the polarizer is perpendicular to the cuvette centerline. The average difference in experimental light intensity between Case A and Case B is 11%, where the uncertainty of light intensity measurements at any given point in the field of view is 4.4%. This uncertainty was established by measuring the variation in observed light intensity at room temperature for constant light source power and in absence of the cuvette. Note that the mathematical model applied for intensity calculations suggests that rotating both filters in 90° should not vary the results. Hence, the experimentally observed differences between Cases A and B may be related to the optical properties of the polarized-light reflector and/or the mirrors in the borescope and polarizer housing (Fig 2).

Fig 7C compares the experimental and simulated light intensity field in case C, where the polarizer is oriented mid-angle between Case A and B. Results in Case C indicate a dependency of the light intensity on the orientation of the polarizer-analyzer couple setup, as is also predicted from the mathematical model applied here. The following observations can be made from Case C: (i) the cavity results in a low light intensity, similar to Cases A and B; (ii) unlike Cases A and B, a region of high light intensity is found at the wall of the cavity, but not close to its tip. A concentrated region of high light intensity is predicted below the tip of the cavity, which is not consistent with experimental results. This disagreement between the experimental and simulation results seems to be arising from reflection at the convex tip of the cavity.

In order to explain the observation that a better agreement between experimental and simulation results is obtained closer to the center of the cuvette, the principal stresses are analyzed along the light paths displayed in Fig 3C, *P*_0_ and *P*_1_. Fig 8 displays the three principal stresses as vectors along these paths. The color scheme in Fig 8 indicates how close the respective stress vectors are to be on a plane defined the path of light as its normal. Here, blue indicates a vector perpendicular to the path of light (i.e., a blue vectors lays on the *x*-*y* plane), while red indicates a vector parallel to the path of light. With this color scheme in mind, at any point for which two principal stress vectors are blue and the third principal stress vector is red, the above assumption that a 3D photoelasticity problem can be analyzed as a series of 2D problems (planar problems) perpendicular to the path of light would be validated. It can be seen from Fig 8 that along *P*_0_ far more stress vectors are closer to blue than along *P*_1_. Indeed, a better match between experimental and simulated results is observed at the center of the domain, as can be observed from Fig 7. The average deviation of stress vectors from the *x-y* plane (excluding vectors in the direction of light propagation) is 4.6% along *P*_0_ and 48.7% *P*_1_. This deviation from the *x-y* plane is calculated as the ratio of the magnitude of the *z* component of the stress vector to the overall magnitude of the vector.

The better match between experimental and simulation results along *P*_0_ could also be explained from theoretical considerations. If the cuvette would be replaced with a long cylindrical vial, the thermal field would be axisymmetric and so would be the resulting thermal stress field [7,31]. In this case, the principal stresses along any path of light passing through the center of the vial would be either perpendicular or parallel to it, and the argument accompanying the interpretation of results associated with Fig 8 would be validated. From similar considerations, and since the cuvette is actually a prism with a square cross section, the cross section of the cuvette has four lines of linear symmetry, along which the first two principal stresses are always perpendicular. The path *P*_0_ in Fig 8A coincides with those symmetry lines, and the principal stresses are perpendicular to it. The only exception in this case is close to the tip of the cavity, which yields distortion of results.

The experimental results displayed in this study were obtained for a cooling rate of 25°C/min. Similar experiments have been repeated at various cooling rates, resulting with light intensity fields, essentially displaying similar trends, where the maximum intensity decreases with the cooling rate. Note that slower cooling rates yielded not only overall lower stress level, but also a shallower cavity, which in turn yielded lower stress concentration.

While the experimental investigation in the current study focused on 7.05M DMSO as a reference solution [48,54,55], CPA cocktails are primarily mixtures of several glass promoting agents, with VS55 and DP6 as examples of cocktails that drew significant attention in the cryobiology community in recent years. Each cocktail results in a unique critical cooling rate to suppress crystallization, and a unique critical rewarming rate to avoid crystal nucleation and/or growth during rewarming (also known as *recrystallization* and *devitrification*, respectively). These cocktails may also vary in their glass transition temperature [48] and mechanical properties [8,50,58,59]. Nonetheless, the constitutive laws describing thermomechanical stresses during vitrification, and especially close to glass transition where the thermomechanical stress is most significant, are essentially the same [48]. Hence, the mathematical modeling described in this study is potentially applicable to a wide range of CPAs, as indeed supported by experimental observations in parallel studies.

In a broader perspective, parallel studies in order to vitrify bulky tissues and organs are now focusing on lower critical cooling and rewarming rates, which are constrained by the underlying principles of heat transfer in large specimens. Reducing the critical cooling and rewarming rates can be accomplished by incorporating special additives to the CPA cocktail, termed synthetic ice modulators (SIMs). The cooling rates demonstrated in the current study may serve as an upper bound to those cutting-edge solutions in the case of large size cryopreservation [56]. Nonetheless, it is noted that the phenomena of (i) structural damage due to thermomechanical stresses and (ii) cell injury due to recrystallization occur in almost completely non-overlapping temperature ranges. However, the physical property that dictates the boundaries of these ranges is essentially the same—the viscosity of the material. Hence, the design of a cryogenic protocol can be informed by thermal stress analysis [7,30,31] and polarized light studies to avoid structural damage at lower temperatures, and by differential scanning calorimetry (DSC) studies to avoid crystallization at higher temperatures [67].

## Summary and conclusions

This study aims at stress analysis associated with polarized light effects during cooling of glass promoting solutions, with applications to cryopreservation and tissue banking. Polarized light means has been integrated previously into the cryomacroscope, a visualization device to detect physical effects associated with cryopreservation success, such as contamination, crystallization, and fracture formation. The current study aims at correlating polarized-light field intensity with thermomechanical stresses, using photoelasticity principles and a polariscope model.

Photoelasticity modeling in 3D in this study is based on subdividing the domain into a series of planar (2D) problems, for which a mathematical solution is available in the literature. The current analysis is based on tracking the accumulated changes in light polarization and magnitude, as light passes through the sequence of planar problems. The current study uses two experimentally fitted parameters, the fringe constant in the CPA and the number of 2D problems the domain is constructed from. The analysis is qualitative in nature, yielding stress behavior and trends, but not the absolute stress magnitude. More broadly, photoelasticity analysis in this study represents a third phase of analysis, building upon an initial phase of thermal analysis, followed by thermal stress analysis.

Results of this study show qualitative agreement in light intensity history and distribution between experimental data and simulated results. The simulated results help to explain differences between 2D and 3D effects in photoelasticity and, most notably, the counterintuitive observation that high stress areas, at times, may correlate with low intensity regions on the recorded image. Therefore, in order to explain stress development in 3D using polarized light, the analysis must be accompanied by modeling and simulations of both the thermal stress field and the polarized light field. For example, it is shown that a better agreement between the simulated results and experimental data is obtained when the magnitude of the third principal stress is relatively small and when the first two principal stress are close to the normal to the path of light. It is also shown that the degree of rotation of the polarization filters with respect to the specimen plays a critical role in photoelasticity analysis.

The results in this study were obtained for a transparent medium, which is a fully vitrified CPA in the absence of tissues or organs. The presence of biological materials would make at least some portion of the domain opaque to a significant degree. Notably, it has been demonstrated in previous experimental studies that the mechanical properties of CPA-loaded tissues in cryogenic temperatures are essentially dominated by the mechanical properties of the vitrified CPA itself [50,54,58]. Hence, conclusions drawn from the currently study in the absence of biological specimens are qualitatively applicable to similar cryogenic protocols in the presence of biological specimens. Here, key similarity parameters include CPA cocktail composition, thermal history, container material, and geometry. Even in cases of opaque media, physical events observed at the container wall [28–30], such as structural damage and fractures, can be used to correlate simulated results with experimental findings. These observations can be used to compare the thermal stress field between the purely vitrified CPA with vitrified tissue-loaded CPA.
